# Supplementary figures and images for: RAB5 NUCLEOTIDE BINDING PROMOTES β-OXIDATION TO FUEL HEPATOCELLULAR CARCINOMA CELL PROLIFERATION
Source: bioRxiv. 2025 Aug 24:2025.08.20.670915. Preprint. [Version 1] doi: 10.1101/2025.08.20.670915 (PMC12393351; doi:10.1101/2025.08.20.670915)

1A

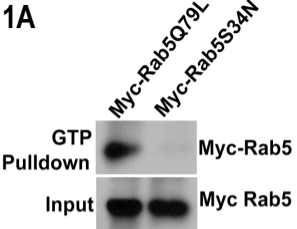

2A

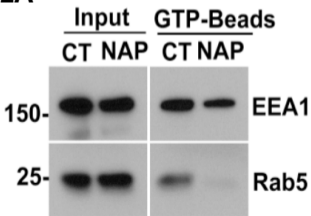

2B

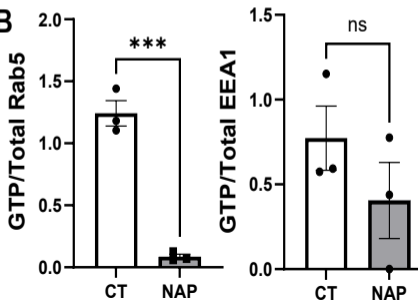

Supplement: Supplement 1 — 1A) Western blot of constitutively active Rab5 (Q79L) and dominant negative rab5 (S34N) GTP-pulldown. To validate that the GTP-bead pulldown works perfectly. 2A) E) Western blot analysis of Rab5 and EEA1 GTP-pulldown in 48h NAP and DMSO control treated condition (nutrient rich media) in Hep3B cell. Note: decrease of Rab5 in the Rab5 inhibited condition (NAP) compared to control (Nutrient rich media). 2B) Quantification of ratio of GTP/total Rab5 in GTP-pull down assay from n=3 independent experiments. Quantification of ratio of GTP/total EEA1 in GTP-pull down assay from n=3 independent experiments. (mean ± SD, p=0.2785, **p<0.005 by unpaired two-tailed t-test). [file media-1.pdf]
